# Access to CKD Care in Rural Communities of India: a qualitative study exploring the barriers and potential facilitators

**DOI:** 10.1186/s12882-020-1702-6

**Published:** 2020-01-29

**Authors:** Tazeen Hasan Jafar, Chandrika Ramakrishnan, Oommen John, Abha Tewari, Benjamin Cobb, Helena Legido-Quigley, Yoon Sungwon, Vivekanand Jha

**Affiliations:** 1 Program in Health Services & Systems Research, Duke NUS Medical School, Singapore, 169857 Singapore; 2 Duke Global Health Institute, Duke University, Durham, NC USA; 3 The George Institute for Global Health, University of New South Wales, New Delhi, India; 4 Saw Swee Hock School of Public Health, National University of Singapore, Singapore, Singapore

**Keywords:** CKD, Barriers, Facilitators, Rural, Qualititative research, India

## Abstract

**Background:**

Despite the high and rising burden of chronic kidney disease (CKD) in South Asia, factors that influence access to CKD care at the community level have not been studied previously, especially in the rural areas. We conducted a mixed methods study and interviewed key stakeholders to explore the views and experiences of key stakeholders, and identify barriers and potential facilitators that influence access to CKD care at the primary care level in rural India.

**Methods:**

A total of 21 stakeholders participated in the study. We conducted 15 in-depth interviews on a purposive sample of stakeholders (CKD patients, healthcare providers and health planners) and one focus group discussion with 6 community health workers. The interviews were audio-recorded and transcribed verbatim. We employed the Lévesque’s framework for access to care to base interview guides and structure the initial codes. By inductive and deductive approaches, thematic analysis was undertaken using QSR NVivo version 11.

**Results:**

The major patient-level barriers to CKD care as reported by the most patients and healthcare providers was poor knowledge and awareness of CKD. Health system-level barriers included shortages of skilled healthcare professionals and medicines, fragmented referrals pathways to the specialists at the hospitals with inadequate follow up care. Many patients and healthcare providers, when asked about areas for improving access to CKD care, reported educational initiatives to increase awareness of CKD among healthcare providers and patients, provision of CKD related supplies, and a systems-level approach to care coordination including task shifting by engaging community health workers in CKD care, as potential facilitators.

**Conclusions:**

We identified several barriers to access CKD care at the primary care level in rural India that need urgent attention. Targeted CKD screening programs and CKD specific educational initiatives may improve awareness of CKD. Additionally, primary care infrastructure needs to be strengthened for CKD care, ensuring trained staff, availability of essential diagnostics and medications, and creating efficient referral pathways for quality CKD care.

## Introduction

Chronic kidney disease (CKD), defined as reduced estimated glomerular filtration rate (eGFR) or presence of albuminuria, is associated with progression to end-stage kidney disease (ESKD), needing dialysis or kidney transplant to sustain life, and increased risks of premature mortality from cardiovascular disease (CVD) [1, 2]. CKD ranked 17th and 8th leading (and one of the most rapidly rising) causes of mortality globally and in India, respectively, by the Global Burden of Disease Study 2016 [3].

Approximately 1 in 5 adults in India has CKD [4, 5]. Diabetes is the single largest contributor to the CKD/ ESKD burden in India, accounting for one-third of the patients with CKD, while other etiologies such as hypertension (13%), glomerulonephritis (14%), and undetermined causes (16%) [6, 7]. The high burden of CKD and associated risk factors have serious implications for a country of 1.35 billion, especially in the rural areas (66.4% of total population in India), where literacy rates are low (65%), and 58% living on less than international $ 3.10 (purchasing power parity) daily [8].

There is strong evidence that the development of CVD and progression to ESKD can be prevented by prompt detection of CKD, and early institution of non-pharmacologic [9, 10] and pharmacologic therapies [11–17]. Since patients with early CKD are often asymptomatic, screening for CKD may improve awareness and health-seeking behavior [18]. Screening and treatment of CKD (albuminuria and eGFR) have been shown to be cost effective in patients with diabetes [19].

However, the health system in India is unable to manage the current and rising burden of CKD, especially in rural areas. Although different cadres of community health workers (CHW) including the auxiliary nurse midwife (ANM) and accredited social health activists (ASHAs) provide basic services related to maternal and child health, and facilitate a link between community and healthcare system (primary health centres), they do not have the mandate or training for health promotion services for non-communicable diseases including CKD. The primary health centres (PHC), each serve a population of approximately 30,000, and most are staffed by only one physician. There is a lower density of qualified doctors in rural India as medical doctors are unwilling to serve rural areas, and PHC is often managed by AYUSH (non-allopathic alternative system) physicians [20, 21]. Further, nephrologists are in very short supply in rural areas in India, as most practicing ones (total of 1850 in a country of 1.3 billion) are concentrated mainly in the urban areas [22]. Shortage and unequal distribution of the healthcare workforce further deter quality care for chronic conditions like CKD.

Although anti-hypertensive and anti-diabetes medications are listed on the WHO essential medication list for government basic health units, these drugs are usually not available in the government primary care facilities. Moreover, significant treatment gaps have been identified with CKD awareness rates being abysmally poor (6%) in India, as in other low- and middle- income countries (LMIC) [1, 23]. Only a minority of individuals with CKD and diabetes achieve recommended treatment targets for blood pressure control (22%) and glycemic control (33%) in India, reflecting poor physician practices and weak health systems [24]. The under-diagnosis and under-treatment lead to high rates of adverse outcomes, including CVD and ESKD. The implications are much worse in rural areas in all South Asian countries where acute CVD event is more likely to be fatal [25].

In addition, there are several social insurance schemes (e.g Employees State Insurance Scheme (ESI) in India, Chief Minister’s Comprehensive Health Scheme in Tamil Nadu, India, however only a small minority (< 20%) of the population have access to these schemes, is mostly for emergency curative treatment at facilities without standardized screening services for CKD or coverage for dialysis. The prohibitive cost of dialysis at US $64 per session in India translates into fewer than 10% of patients with ESKD receiving renal replacement therapy, and thus the vast majority die prematurely [26].

More recently the National Programme for Prevention and Control of Cancer, Diabetes, Cardiovascular Disease and Stroke (NPCDCS) has been introduced as a pilot program in selected states where the ANMs are expected to screen adults for diabetes and hypertension at non-communicable diseases (NCD) screening camps. However, this programme has also neglected CKD.

Evidence is mounting regarding the role of trained non-physician health workers in the management of hypertension and diabetes in South Asia [27–29]. Furthermore, digital platforms are being increasingly used for health promotion, as well as screening and management of non-communicable diseases [30]. However, the factors that influence access to early-stage CKD care in rural communities of India and neighboring countries are yet to be studied [31]. Understanding the challenges faced by the patients and providers regarding the management of early CKD is critical to designing strategies that are potentially effective for improving outcomes.

Our qualitative study aimed to explore the experiences and views of key stakeholders (i.e. CKD patients, healthcare providers, and health planners) regarding factors influencing access to care for CKD in rural communities of India. The primary objective was to understand the barriers and potential facilitators to CKD care at the primary care level. The ancillary aim was to discern the perceived usefulness of a mobile-technology based clinical decision support system (mCDSS) for CKD care in the primary healthcare setting.

## Methods

### Study setting and design

The qualitative study was embedded within the Innovative M-health led Participatory Approach to Comprehensive Screening and Treatment of Diabetes study (IMPACT Diabetes study); which aimed to test the feasibility and acceptability of a comprehensive mCDSS based intervention for community-based diabetes management, in the PHC areas served by the Pandit BD Sharma University of Health Sciences, Rohtak, Haryana, India. Four PHCs were selected based on convenience (accessibility of PHC and availability of the PHC physician). Within each PHC, two villages -one big (~ 6000 population) and other small (~ 3000 population) were randomly selected from all the villages being served by the PHCs. The study population included stakeholders- a) adults (> 18 years of age) male or female patients with confirmed CKD attending renal clinics in the study area for at least 3 months irrespective of stage of the disease; b) healthcare providers- namely nephrologists, primary care physicians working in the renal clinic at the time of study, and frontline community healthcare workers (ANM and ASHA) from the study PHC areas involved in screening, referral and management of CKD patients; and c) health planners comprising of government officials from the state ministry of health responsible for NCD programs. Respondents were selected by purposive sampling, specifically selecting frontline healthcare workers and doctors, targeting 3–5 individuals in each stakeholder category.

We developed the interview and focus group discussion (FGD) guides with open-ended questions to solicit the participants’ experience and views concerning CKD care in rural communities. We adopted the Levesque et al’s framework to design our interview guides and collect the data [32]. The guides covered topics such as knowledge and awareness of CKD, current preparedness and practice for the management of CKD, facilitators of and barriers to CKD care, and perceived usefulness of mCDSS in CKD care and management.

Pre-testing was undertaken among representative respondents from individuals with CKD, healthcare providers, and government stakeholders from the study area who participated in another implementation research project. The pre-testing consisted of administering the interview schedules and recording the responses. Responses were then reviewed by an independent researcher, and the interview schedules were modified to include contextually relevant questions and sequence of the open-ended interview schedules.

The researchers developed an in-depth understanding of the barriers and potential facilitators to CKD management by spending time with study respondents and eliciting responses to cover all the key aspects of the interview schedule. It was important to spend the initial minutes of the interviews to build rapport and gain the confidence of the participants. In cases where respondents were under time-constraint, the interviews were rescheduled so as to avoid poor quality response. This was particularly the case with physicians and government officials. While at least five participants were approached in each category, the availability of doctors (primary care physicians), nephrologists, and government officials were constrained due to competing priorities. One nephrologist and two primary care physicians were included as respondents, and we grouped the clinical care providers into one category as physicians for ease of analysis of the themes from the clinical care delivery perspective to identify health systems-level barriers. Total 15 one-to-one interviews were conducted but at initial data analysis of the one-to one interviews, data saturation in some of the framework’s dimensions (availability and accommodation) were not reached. Therefore, we conducted one FGD additionally with frontline community health workers, ASHAs (*n* = 6), in the study areas to complement the data until data saturation was achieved.

Following approvals from departments, researchers visited field areas and renal clinics for data collection. For healthcare providers (physicians and community health workers), appointments were secured and interviews scheduled in break time to avoid interruption of services. The moderators collected demographic information, followed the interview /FGD guide, and asked open-ended questions. Additional file 1 provides a synopsis of interview guides. The one-to-one interviews lasted between 20 and 30 min while the focus group lasted approximately 60 min, and were conducted by AT and OJ. Each selected respondent was interviewed in a private room, and in a quiet location at the premises of the healthcare facility or local research office. The interviews were conducted in either local language (Hindi) or in English, audio-recorded, and transcribed verbatim. Transcripts were checked to ensure that they did not contain any mistakes made during transcription. For confirmability, the researchers were careful in not allowing prior knowledge of the patients’ condition to affect the way in which the interviews were conducted. The transcripts in Hindi were subsequently translated into English by bilingual interviewers and reviewed by the research team.

**Fig. 1:**
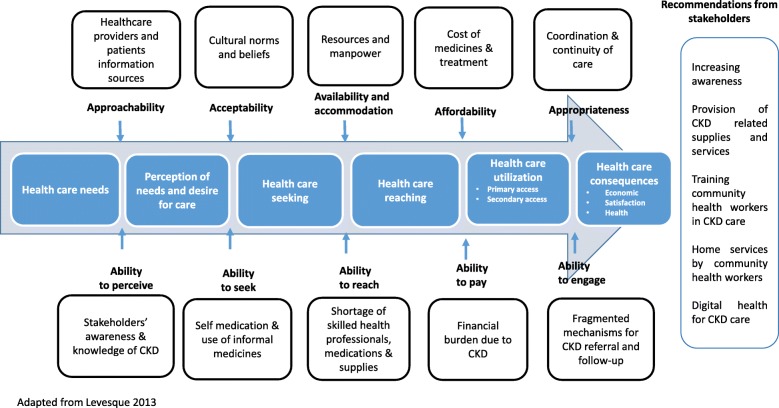
Conceptualization of access to CKD care through stakeholder perspectives from rural communities of India

### Data analysis

We employed both the grounded theory approach and conceptual modeling underpinned by Levesque et al’s framework [32] to collect and analyse the data. We analysed all the interviews and FGD transcripts thematically [33]. The search for themes began by reading and immersing within a single transcript to draw preliminary interpretations. A list of emerging themes and their relationships allowed the themes to be grouped together as master themes. A code was assigned to each theme using QSR NVivo 11 software. The list of master themes was then compared to those generated through the remaining transcripts. This process allowed themes and explanations to arise inductively from the data. All themes were simultaneously mapped against the Levesque et al’s framework to denote data alignment with the framework’s conceptual elements and identify new themes developed inductively. Two research team members (CR, SY) independently coded a subset of data and compared coding. The consensus was reached through discussion and iterative review of codes and categories. This involved a process of constant comparison of between- and within- categories, and refining and recoding of the text until an array of interlinking themes was elicited. All codes were then reviewed together by the research team (THJ, CR, OJ, AT, BC HLQ, SY, VJ) to ensure that common themes reflected a shared understanding among participants of the phenomena under investigation. In addition, quality assessment checks for coding were performed on 20% of randomly selected transcripts by THJ. Data saturation was achieved, with no new themes emerging from the data (see Additional file 2 for the Consolidated Criteria for Reporting Qualitative Research-COREQ).

### Conceptual framework

We used Levesque et al’s access to care model [32] as a conceptual framework to understand factors influencing access to care at the health systems and population levels. The five dimensions of the framework include; 1) approachability (ability to perceive); 2) acceptability (ability to seek); 3) availability and accommodation (ability to reach); 4) affordability (ability to pay); and 5) appropriateness (ability to engage). Moreover, interaction among the different set of dimensions needs to be accounted for when planning to operationalize the framework.

## Results

A total of 21 stakeholders participated in the study. More than half of the participants (62%) were females. Among 14 healthcare providers (HCPs), 11 were community health workers (included ANM and ASHA). Five patients and two district-level officials also participated (Table 1).

**Table 1:** Characteristics of participants

		Gender	
Category of stakeholder	N (%)	Male	Female
Healthcare provider *n* (%)	14 (66%)	1	13
*Community health workers*	*11*	*0*	*11*
*Physicians (2 Primary care physicians & 1 nephrologist)*	*3*	*1*	*2*
CKD Patients *n* (%)	5 (24%)	3	2
District level official *n* (%)	2 (10%)	2	0
Total *n* (%)	21 (100%)	8 (38%)	13 (62%)

The key themes as per Levesque et al. access to care framework dimensions are presented in Fig. 1

### Approachability and ability to perceive: stakeholders’ awareness & knowledge

Approachability and ability to perceive refers to the healthcare providers’ and patients’ opportunities to identify CKD services that exist and can be reached [32]. Tied to this approachability concept is awareness, which relates to the ability to perceive the need for care. The important themes identified as barriers and facilitators to approachability and ability to perceive were:

#### Barriers

##### Poor knowledge & awareness of CKD among HCPs and patients

A common theme across participants’ accounts was poor knowledge and awareness of CKD. As one healthcare provider reported, there was a general “lack of awareness among patients and even doctors.” Primary care physicians reported having limited knowledge and confidence in managing early CKD. Although the primary care physicians were familiar with the terminologies such as “urea” and “creatinine,” they did not proactively screen for CKD, nor did they manage patients diagnosed with CKD, rather the latter were referred to specialist centres.

Likewise, the CHWs had low awareness of CKD in terms of the risk factors, and detection and complications of CKD. They had the misconceptions that CKD screening required multiple tests that were not available in the primary care setting. Since the CHWs’ existing job scope centred on mother and child health and communicable diseases, it further constrained them from providing CKD-related services.

##### Low-risk perceptions among patients resulting in delayed diagnosis

Overall, most of the stakeholders perceived that the burden of CKD had increased over the years, and “many people around them are suffering from ESKD.” Many healthcare providers reported a rising prevalence of diabetes “in the villages.” Nonetheless, the perceived increase in the prevalence of diabetes did not translate into the uptake of screening for CKD, and most patients were not aware that diabetes was a major cause of CKD. The above-mentioned poor perceived risk of CKD appeared to contribute to delayed diagnosis of CKD, with participants recounting experiences from family or friends who were “diagnosed late” when the kidney was completely damaged.

##### Inadequate patient-provider communication regarding CKD

Some patients expressed that they received insufficient information on CKD from healthcare providers, which undermined seeking and acquisition of knowledge. Conversely, healthcare providers frequently identified patients’ low health literacy and acceptance of CKD screening and treatment as major challenges for effective communication.

#### Potential facilitators

##### Increasing awareness of CKD

Most participants strongly expressed the need to increase CKD awareness amongst both healthcare providers and patients. A “right place” and “right people” strategy was suggested to improve awareness. Most stakeholders recommended partnering with Anganwadi centers (rural centers for maternal and child programs) and schools to conduct screening and dovetailing diabetes and NCD awareness initiatives with established educational programs such as the information, education, and communication (IEC) of the World Health Organization, which presents an opportunity to improve awareness. Furthermore, outreach camps at “convenient” locations like the Anganwadi centres, mass media campaigns, and dissemination of printed pamphlets were also suggested by some as viable options for CKD health education.

Although participants opined that ASHAs could provide CKD education during their home visits, some felt this could be time-consuming, and could take valuable attention away from regular patient care activities.

### Acceptability and ability to seek: cultural norms

‘Acceptability and ability to seek’ refers to the cultural factors and norms that influence how populations accept the aspects of services provided [32]. An important sub-theme identified in this domain was:

#### Barriers

##### Self-medication and use of informal medicines

Many healthcare providers reported that patients’ cultural beliefs and norms were often at odds with their clinical recommendations, thereby creating challenges with the management of CKD. The providers cited the use of alternative medicines by the patients to treat diabetes and CKD as one of the major barriers to the provision of quality CKD care. Indeed, patients reflected on self-medication or seeking non-traditional treatments from complementary medicine practitioners to treat their chronic health conditions.

### Availability and ability to reach: resources for CKD care at primary care level

‘Availability and ability to reach’ refers to the existence of health services for CKD [32] and is shaped by the availability of facilities and health resources. The sub-themes identified in this dimension were:

#### Barriers

##### Inadequate human resources

Many providers and government officials reported that primary care was largely directed towards maternal and child health, and thus “very little” resources were available for NCD. Although the basic management of diabetes was generally perceived to be sufficient, however comprehensive care including screening for microvascular complications was perceived to be inadequate primarily due to the lack of resources, including shortage of skilled and trained providers. Consequently, CKD patients were most often referred to the district hospitals. PHCs were unable to cope with the patient load. Most patients indicated being burdened from the general frustration of “staff shortage all the time”, and recounted experiences of crowding and long waiting times at the PHCs.

##### Shortage of medicines and diagnostic supplies

Many healthcare providers and patients reported issues related to the availability of medicines and diagnostic supplies related to CKD at PHC. The healthcare providers attributed the shortage of resources to increased patient load, while government officials expressed that there was occasional “disarray” in medicine supply. The scarcity of medications often resulted in patients having to purchase medicines out of pocket.

#### Potential facilitators

##### Provision of CKD related supplies and HCP training

Most HCPs and patients expressed a strong need for improving CKD services and ensuring the availability of medicines, tests, and doctors including nephrologists at the PHC. Primary care physicians voiced the need for supplies and resources for CKD screening tests to be made available at PHC. In addition, some physicians also suggested dedicated clinic days for screening and evaluation of family members or friends referred by the CKD patients. Additionally, the need for training of primary care providers in CKD management was mentioned by a nephrologist.

##### Home visits by trained community health workers for CKD care

A theme running through the data was task shifting to ASHA to facilitate CKD care in rural communities. Patients recognized that home visits by ASHAs for CKD will be advantageous and minimize the inconvenience of traveling long distances for regular blood checks.

Many ASHAs were amenable to performing CKD related tasks during home visits and desired greater degree of involvement in the care of patients with chronic conditions. However, some expressed “a bit of fear” since they had no prior experience with CKD, and were apprehensive about “increase in workload” and “lack of time” HCP and government officials suggested the need for “skills training” for ASHAs. ASHAs were largely acceptable to the idea of vocational training, which, they felt, would empower them to provide relevant advice to patients.

### Affordability and ability to pay: cost of medicines and treatment

Ability to pay refers to the economic capacity of people to spend resources and time [32]. With respect to CKD, the sub-theme emerged were:

#### Barriers

##### Financial burden due to CKD

Some healthcare providers believed that due to the patients’ fear of high cost, timely preparation for RRT was not feasible. It was commonly perceived that the poor are “unable to afford” treatment, and that “financial problems [associated with treatment] would break down poor man”. One patient undergoing dialysis voiced the need that “patients should get financial support”.

### Appropriateness and ability to engage: continuity of care

‘Appropriateness and ability to engage’ refers to the fit between services available and patient needs [32]. Tied to this domain are adequacy, quality, and system integration, which ensure continuity of services, and influence the ability to engage. The key findings in this dimension were:

#### Barriers

##### Inadequate mechanisms for CKD referral and follow up

Primary care physicians reported “referring” all patients with CKD to the specialists because the rural PHCs could not offer services for CKD. Although referral registers are maintained, there was lack of a mechanism for follow up of patients in primary care, which depended entirely on the patients if they visited the PHC “by themselves”. As reported by most patients, “there is no mechanism for follow up”.

Healthcare providers stated that the referral process for patients with ESKD needing RRT was considerably delayed due to difficulty in obtaining a specialist appointment. They mentioned that these delays in referral were associated with a shortage of beds in the hospitals relative to the high demand. Consequently, the waiting times for patients with ESKD to receive RRT were long. In addition, the problem of “distance” to receive treatment was reported as a barrier to CKD care by some and caused considerable dissatisfaction. There was no formal mechanism of communication between community healthcare workers and nephrologists.

#### Potential facilitators

##### A system approach to care coordination

Healthcare providers recommended that a “system should be there” where patients with CKD are examined and appropriately referred to specialist. The primary care physicians reported that the education of doctors, and providing a systematic “follow-up” of referred patients was important for continuity of care, as these measures could support CKD care and improve medication adherence. Additionally, such a coordinated system could enable better patient satisfaction, if widely available, could improve CKD care.

##### M-health technology to improve CKD care

Most participants were supportive of m-health approach to CKD care. Participants perceived m-health to be “convenient,” “beneficial’, “feasible, and offers benefits of care at the doorstep saving time and resources.” Other perceived positive attributes of m-health technology were the potential to address gaps and enable sharing of records, patient information, and timely treatment.

However, some participants foresaw the complexity of implementing m-health due to “slow” internet connectivity and ‘illiteracy’ in rural areas. Some stated that m-health support would be futile if patients do not read the messages, and therefore needs to be tailored to the local language. One government official disagreed with the m-health approach voicing that efforts should be prioritised on addressing health systems barriers to ensure the community has access to quality primary care.

Table 2 provides a summary of themes, subthemes, and illustrative quotes.

**Table 2:** Summary of themes, subthemes with illustrative quotes

Key themes & subthemes	Evidence
Approachability: Stakeholders’ awareness & knowledge of CKD	
*Barriers*	
Poor knowledge & awareness of CKD among HCP and patients	*“Have little bit [of] knowledge in kidney disease because they [primary care physicians] do not know much beyond the urea, creatinine, because the reason behind is that once the kidney disease has been diagnosed, we refer them. We do not manage it at PHC level. If we manage, then our juniors will also learn.*” Physician 1*,* Male *“Actually we [patient & wife] did not know that this was a kidney problem. I had breathlessness, then we [patient & wife] came here [hospital] and doctor did some test. He found that creatinine is high then KFT was done, it was high, then doctor asked to get it [condition] treated, then he came to the kidneys, gave medicines, and pulled me for 2 years*.*”* Patient 4, Male
Low risk perceptions among patients resulting in delayed diagnosis	*“This was the problem with my sister in law, she had frequent problem in going to toilet, started bleeding in the toilet. As she went to treatment, they came to know that one of her kidney is completely damaged, and operation date was been fixed for renal transplant. But, she got expired 2 days before the operation was supposed to be done”-* Community health worker (FGD participant), Female *“I had weakness and got hypertension in 2008. In September 2009 I lost weight suddenly. I thought that this would have happened because of some stress, but that was the first sign, which I had ignored. In March–April 2010, I started having lot of problems, like breathlessness, anxiety, I was unable to sleep. By May 2010, when problems increased, I got my test done, and in that creatinine, urea, etc were increased, it [CKD] was confirmed and I came to know like this”.* Patient 5, Male
Inadequate patient-provider communication regarding CKD	*They [doctors] said that your kidneys have a problem, and other than this they [doctors] did not say anything.* Patient 1, Male
*Potential Facilitators*	
Increasing awareness of CKD	*“Yes they [patients] should be given information! Until the public will not be aware, how will they know?”* Community health worker 2, Female *Yes, it should be done, awareness should be spread, like for a patient or someone normal, they will know about their disease that what are the symptoms of disease, and then they [patients] will take more care and will go for continuous routine checkups. They must have awareness”* Patient 2, Male
Acceptability: Cultural norms & beliefs	
*Barrier*	
Self-medication & use of informal medicines	“*People don’t prefer going to a nephrologist. Rather they would be told by someone to have indigenous medicine, or if it is kidney disease, if somebody else consumed soda, and few days after drinking soda, the results come, in that 25% of his kidneys have stopped functioning.”* Community health worker (FGD participant), Female *“My father takes medicine, it is Chandrprabha (Ayurveda medicine) and second one is capsules of defit. There was a program of MI [name] company, medicines came [bought] from there costing 5500–6000[Rupees], we have purchased it. My father takes it but I don’t.”* Patient 2, Male
Availability: Resources and manpower for CKD care at primary care level	
*Barriers*	
Inadequate human resources	*Like for chronic kidney disorders, at present in some centers, it’s [tests] not started yet, although it is going to be operational, the machine has been seen, there is a problem of AC or something, I exactly don’t know much. But it is not functional as it has to be kept in the AC [air conditioner]. The machine is needed to do test for creatinine, urea,* etc. *So, accordingly screening of chronic kidney disorders can be done here and also for blood glucose. So it will be done. Just the same problem remains of manpower shortage.* Physician 1, Male “*For the test madam the staff is less. The main thing is of staff. If staff is complete, then there will be no problem, if the staff is less, then it [PHC] becomes crowded.”* Patient 2, Male
Shortage of medicines & diagnostic supplies	*“Our calculations are sometimes mismanaged, because we have to indent first. We order the medicines by indenting but if patients are increased then there is shortage of medicines*.*”-* Physician 1, Male *“I go to doctor for check-up once in a month. He monitors weight, blood pressure and gives me the same medicine. Sometimes I buy it from market and sometimes he gives me.”* Patient 3, Female
*Potential Facilitators*	
Provision of CKD related supplies and HCP training	*“For that, at all PHCs and CHCs, treatment should be available there also. As for injections, tablets should be there, a pharmacist who should be available there 24 h.”* Patient 2, Male
Home visits by trained community workers for CKD care	*“Our knowledge should be increased, like what sugar is and what happens if it increases. Thee more information [as part of training] is given it is better. I want to get further knowledge so that we can give it anyone else and it will be beneficial. “*Community health worker 4, Female
Affordability: Cost of medicines & treatment	
*Barrier*	
Financial burden due to CKD	*“They [dialysis patients] are unable to understand that what is happening, one such disease has happened and above that it costs 50,000 per month. From here begins the frustration of human, if someone can work on this then I think 90% of the problems will be solved. Most of the families are unable to come for dialysis three times a week. Patient comes only once a week, they have to take protein diet but they are eating pulse and rice only. I am living here in Delhi just for treatment. I came here for the transplant, but that did not happen and now I am on dialysis. So I am staying here in Delhi, transportation, food, dialysis, all costs a lot.”* Patient 5, Male
Appropriateness: Co-ordination and continuity of care	
*Barrier*	
Inadequate mechanisms for CKD referral and follow up	*We have a general OPD [out-patient department] register. It has separate part for the referral, like how many referrals had been done, how many are done here only. This way it is managed. Projects are being run by the government and if we have to notify something then we mention them separately and report separately. Everything else is done in the General OPD and for referrals, like we are unable to manage it (CKD), we refer them to General hospital.* Physician 1, Male
*Potential Facilitators*	
A system approach to care coordination	*“For their satisfaction they can get the facility around their area, because the district level becomes quite crowded. Even if any program runs for this, then we can follow-up them as well, at the primary level and secondary level also. There follow-up will be done in our area only, then they will not have to run here and there or shift anywhere else.”* Physician 2, female *“System should be there where a kidney patient referred is being checked by MO [medical officer] sir and referred further accordingly”* Community health worker (Participant 4-FGD), Female
M-health technology to improve CKD care	*So, if you have a mobile app or some software in the computer and if you train them, how to use that it will be really helpful because a PHC doctor has to do a lot many activities apart from the clinical work. They do a lot of managerial work and time is very limited. So 1 day NCD people will they present them as NCD nodal officer, the next day RNTCP team come they represent as RNTCP person so he plays different roles. So if you assist him with a properly guided come portal in the form of a mobile app or a software, I think (in my personal opinion) they will be happy to have that”* Government official 1, Male *If it [m-health technology based care] will be provided then people and anybody naturally will be benefitted.”* Patient 4, Male

## Discussion

This qualitative study identified key barriers for access to CKD care among rural communities of India, which if addressed effectively, could potentially avert several negative health, social, and economic consequences associated with advanced CKD.

Our findings suggests that while some patients reported serious concerns about rising burden of CKD in the communities, the majority, had low awareness of its risk factors, adverse consequences, and on how to prevent or treat CKD. The lack of motivation for screening and management could be explained by the absence of symptoms of early stages of CKD and low perceived risk of CKD. Moreover, the current process of referrals of patients with symptoms of CKD to the specialists have issues of long travel distance, waiting time, and bureaucratic complexity, and needs to be streamlined to enhance efficiency.

Studies from other regions of the world have reported poor knowledge and awareness to CKD amongst healthcare providers and patients which aligns with our findings [34, 35]. [36] Experiences of delayed diagnosis of CKD among our rural community participants are concordant with perspectives and experiences on CKD from marginalized groups [37]. Recent large multinational surveys by the International Society of Nephrology (ISN) reported similar patient-related factors – knowledge, attitude and geography, and physician-related factors - availability, access, knowledge and attitude as barriers to optimal kidney disease care in South Asia [36].

The shortage of medications and supplies is an additional key barrier to CKD care in rural India. This is unsurprising as the ISN survey showed that only 30% of LMIC had access to health technologies like measurement of serum creatinine and urine albumin testing, none had access to eGFR and quantitative estimation of albuminuria, and low availability of essential medications for kidney disease [38, 39]. Our findings underscore the need for making the very basic diagnostic supplies for CKD (urine protein dipsticks and measurement of serum creatinine), and anti-hypertensive, glucose-lowering and lipid-lowering medications accessible to patients with CKD. This approach would be consistent with universal health coverage, and assist with achieving the Sustainable Development Goal 3.4 to reduce by one-third premature mortality from NCDs through prevention and treatment. Furthermore, given our findings of frustration expressed by the patients over the need to navigate several layers of bureaucracy in order to access to a specialist, there is an urgent need to streamline the referral process and create efficiency in CKD in part by the introduction of strategies for patient activation and empowerment. Patient activation could potentially help improve self-management behaviors and health outcomes [40].

Increasing CKD awareness was recommended as a potential facilitator for improving access to CKD. Educational initiatives courses and modules on CKD for primary care physicians have been shown to increase knowledge regarding CKD and could be adapted for primary care setting in India [41, 42]. Targeted programs like the Kidney Early Evaluation Program (KEEP)- a targeted community screening program for CKD in individuals with high risk of CKD has shown to improve awareness of CKD, and in-turn health-seeking behavior of the population [43]. Screening for CKD has been shown to be cost effective in diabetes in HIC [17, 19, 44]. While similar evidence is needed from LMIC, given the high prevalence of CKD and associated premature mortality and the unaffordability of dialysis, CKD screening is likely to offer even more economic returns on the investment.

Preventive strategies centered on non-physician health workers have been shown to be effective for control of hypertension and diabetes [27, 45]. Innovative models of collaborative care with primary care physicians and training non-physician health workers in CKD care could improve quality of services, continuity of care and address the shortage of nephrology workforce in LMIC.

The World Health Organization Package of Essential Non-communicable Disease Interventions promises hope for CKD prevention. To be fully effective, such strategies should focus on individuals with high-risk of developing CKD, such as those with diabetes, hypertension, family history of CKD or exposure to environmental factors, such as manual work in hot and humid environments (heat stress nephropathy) [46], or local customs, such as consumption of traditional medicines and over the counter use of non-steroidal anti-inflammatory drugs. Such high-risk individuals should be followed by providing guideline-based care to those who screen positive, reducing non-adherence to therapy, and instituting quality improvement programs for the management of CKD. Comprehensive control of CKD would involve a collaborative model of care starting from screening and identification of early stage disease, continuing through to end-of-life support for those with advanced disease [47]. Novel m-health tools for care support, endorsed as beneficial, acceptable, and feasible could help strengthen health services delivery for NCD, and such tools need further evaluation for use in resource-limited settings.

### Strengths and limitations

To our knowledge, this is the first qualitative study from rural India, exploring factors influencing access to early CKD care. A key strength of this study was its inclusion of a wide range of stakeholders in the health system spanning health system leaders, community care workers, and patients, which enabled us to explore various experiences and perspectives regarding CKD care in rural communities of India. The inclusion of various stakeholders not only brought greater clarity to the factors influencing CKD care, but it also allowed triangulation of data grounded in stakeholders’ experiences. In addition, drawing on the Lévesque’s framework [32], we employed both inductive and deductive approaches to generate a nuanced understanding of the access to CKD care and how these challenges can be addressed. Our analysis has demonstrated that various dimensions of the Levesque’s framework were highly relevant to holistically understanding access to CKD care in low resource settings.

Our study also has limitations. The small sample size in each group of stakeholders may have diluted the views of the stakeholder group. Due to the shortage of physicians in rural communities, only three physicians participated in the study. It is, therefore, possible that limited representation of different cadres of health professionals may have influenced the themes and suggestions generated in this study. However, this limitation was counteracted by purposively recruiting more CHWs to ensure that the results of the study represent the perspectives and inputs from healthcare providers working in the field. Similar surveys among a larger sample of primary care physicians in India and other South Asian countries is needed. Furthermore, only patients with CKD due to diabetes were included. However, diabetes is the commonest (44%) cause of ESKD in India [7], and the challenges faced in accessing care are expected to be similar for patients with other causes of CKD. Moreover, since our participants were recruited from a few selected villages in North India, findings may not be generalised to all rural communities of India. However, similar findings regarding poor awareness and weak healthcare services have been reported for hypertension management from other countries like Bangladesh, Pakistan, and Sri Lanka [48]. Thus we believe our findings on CKD would be generalizable to other countries in South Asia and possibly many LMIC.

## Conclusions

This qualitative study demonstrates poor awareness and knowledge on CKD among primary care providers and patients, and unprepared primary care infrastructure to be the key barriers for access to CKD care in rural communities in India. There is an urgent need to address the system-level barriers to CKD care by increasing the awareness among primary care physicians and patients, engaging community health workers, improving supplies for diagnostics and medications for CKD in the primary care clinics, and creating efficient referral pathways. Further research incorporating m-health tools to enhance and support CKD care in diabetes could be evaluated. Such strategies could provide an opportunity to address the gaps and strengthen health services delivery in CKD care.

## Supplementary information

**Additional file 1:** Summary of interview guides.

**Additional file 2:** Consolidated criteria for reporting qualitative studies (COREQ): 32-item checklist.

## Data Availability

The datasets used and/or analyzed during the current study are available from the corresponding author on reasonable request.

## Abbreviations

ANM: Auxiliary nurse midwives
ASHA: Accredited social health activists
CKD: Chronic kidney disease
CVD: Cardiovascular disease
eGFR: Estimated glomerular filtration rate
eSKD: End-stage kidney disease
FGD: Focus group discussion
HCPs: Healthcare providers
IEC: Information, education, communication
IMPACT: Innovative M-health led Participatory Approach to Comprehensive Screening and Treatment
ISN: International Society of Nephrology
KEEP: Kidney Early Evaluation Program
LMIC: Lower- and middle income countries
mCDSS: Mobile-technology based clinical decision support system
mHealth: Mobile-health
NCD: Non-communicable diseases
NPCDCS: National Programme for Prevention and Control of Cancer, Diabetes, Cardiovascular diseases and Stroke
PHC: Primary Health Centres
RRT: Renal Replaement Therapy
