# Supplementary material for: Access to CKD Care in Rural Communities of India: a qualitative study exploring the barriers and potential facilitators
Source: BMC Nephrol. 2020 Jan 29;21:26. doi: 10.1186/s12882-020-1702-6 (PMC6988353; doi:10.1186/s12882-020-1702-6)
Supplement: Supplementary file 1 — Additional file 1: Summary of interview guides. [file 12882_2020_1702_MOESM1_ESM.docx]

**Additional file 1: Summary of interview guides**

| **Topics** | | | |
| --- | --- | --- | --- |
| **Stakeholder category** | **Existing knowledge & current practices on DM)/CKD** | **Barriers & facilitators for CKD management** | **Perceived usefulness of mobile-clinical decision support system (mCDSS) for CKD management** |
| **Government officials** | What are the efforts from the government for NCD? Is there a separate DM/CKD control program?  What are the current diabetes and kidney diseases related programmatic initiatives by the Government of India?  Where are these programs being implemented currently states and urban/rural  What health service standards are currently established and agreed upon by the MOH for the treatment of DM/CKD, if any? | What are the main challenges for quality DM/CKD management in India and particularly in the rural areas? How do you think you can overcome the problem?  How is the overall health delivery for providing quality DM/CKD care to the rural population?  What do you think will enable better provision of quality DM/CKD management? | Do you think a diabetes and CKD management program using mCDSS will be helpful in rural communities?    What do you think is the preparedness of the PHC for accommodating a CKD management program using mCDSS-SMART health will be helpful in rural communities? What will be the challenges?  Do you think that there will be challenges for integration into the Primary Health Centre (PHC) system? If yes, what challenges? If no, why not?  Do you think there will be challenges for scalability of such a program? If yes, what challenges? If no, why not? |
| **Nephrologist** | What are the current standards of care for NCD like DM/CKD from your perspective?  Could you describe your current work practice?  How do you currently manage patients with CKD?  What are the current referral practices for sending patients? How are the patients with DM/CKD referred to you i.e. the mechanism of referral?  What is the approximate rate of people who receive a follow-up that actually end up going to the doctor to which they’re referred? If they do not go, do they go to an alternate doctor or not go at all? | What do you think will be the factors that will encourage better DM/CKD management in rural areas?  In general, do you feel there is a need to change how DM/CKD is currently managed in your district?  What are the main challenges for DM/CKD management in your district/area?  What do you think will be the factors that will encourage better DM/CKD management in rural areas? Which one is the most important? Why?  What do you think about integrating DM/CKD screening & referral into the work practices of ASHA? Why do you think it is helpful or not? | Are you aware about using mobile technology approach to managing chronic disease?  Have you heard about clinical decision support system (CDSS)?  CDSS will be based on an algorithm for management of diabetic CKD. What do you think is important in the CKD management algorithm?  Do you think a diabetes and CKD management program using mCDSS-SMART health will be helpful? |
| **Primary care physicians** | What are your current duties as a PHC doctor?  What are the work practices of PHC doctors in relation to DM/CKD management?  Do you get some training in managing DM/CKD management?  What is the mechanism of referral to the next level of care (nephrologists) for DM/CKD complications? | What do you think will be the factors that will encourage better DM/CKD management in rural areas?  In general, do you feel there is a need to change how DM/CKD is currently managed in your district?  What are the main challenges for DM/CKD management in your district?  What do you think about integrating DM/CKD screening & referral into the work practices of ASHA? Why do you think it is helpful or not?  .  Do you think that DM/CKD management will this impact other services provided by the PHC doctors? If yes, why? If no, why not? | Are you aware about using mobile technology approach to managing chronic disease?  Do you think diabetes and CKD management program with aid of a mobile tablet device will be helpful to PHC doctors?  What do you think will be the difficulties encountered during such a mCDSS DM/CKD management program in rural areas? |
| **Community health workers** | In general, what are the main duties/priorities of accredited social healthcare activists (ASHAs) currently?  What are the current work practices of ASHA in relation to DM/CKD management in the community?  What are the current attitudes/levels of knowledge of ASHA on DM/CKD management? | What do you think of home diabetes/CKD screening in your village?  What will be the difficulties you will face with regards to time and incentive during the home based screening and education sessions into your current work schedule?  Do you think this work will impact other services? What are your plans to ensure that other services are not impacted? | Are you aware of mobile health way of managing chronic diseases?  The ASHA will use the tablet with alerts and scripts available for screening, referral and home health education. Do you know how to use tablet devices and mobile devices?  What do you feel will be the usefulness of such mCDSS system from your view?  What will be the challenges or difficulties encountered in such a DM/CKD program? |
| **Patients** | Can you describe how your doctor has been checking you for diabetes/CKD?  What does your doctor tell you or discuss about your diabetes/CKD during the clinic visits?  What do you find easy/ difficult about managing your DM/CKD?  What do you think could be improved in terms of the care you receive for your DM/CKD? | What services do ASHAs currently provide your household?  Have you, or any member of your household, ever had your blood glucose and/or BP and/or kidney function checked?  What do you think are the benefits for you if ASHAs were to check your blood sugar/BP/kidney function for screening for diabetes/CKD in your home?  Do you have access to medications for diabetes/CKD? | What do you think of a program based on mobile technology for DM/CKD?  Do you think it would be beneficial if you were given SMS to remind you about the follow up doctor visit for tests and review?  What will be the challenges or difficulties encountered with such a program? |
| **Focus group discussion guide -Community health workers** | According to you what is chronic kidney disease?  Have you ever received any training in providing services for CKD care?  Please tell us about the available services for CKD screening in your area?  How do the patients with CKD pay for the treatment? | If you are trained in drawing blood and given additional responsibility of testing blood and urine albumin for CKD, what will be the difficulties (barriers) you will encounter?  With the above-mentioned additional activities, will this be practical for you to manage your existing duties?  How can the above activities be facilitated? | If we were to start a program and provide services for CKD, who all can be part of the program?  In such a program, how will the interaction be between the members like PHC doctors, district level managers etc?  Please suggest how can we overcome the problems |
